# Self‐Assembly of Heterogeneous Ferritin Nanocages for Tumor Uptake and Penetration

**DOI:** 10.1002/advs.202309271

**Published:** 2024-02-17

**Authors:** Qiqi Liu, Chunyu Wang, Mingsheng Zhu, Jinming Liu, Qiannan Duan, Adam C. Midgley, Ruming Liu, Bing Jiang, Deling Kong, Quan Chen, Jie Zhuang, Xinglu Huang

**Affiliations:** ^1^ State Key Laboratory of Medicinal Chemical Biology Key Laboratory of Bioactive Materials for the Ministry of Education College of Life Sciences and Frontier of Science Center for Cell Response Nankai University Tianjin 300071 China; ^2^ Nanozyme Medical Center School of Basic Medical Sciences Zhengzhou University Zhengzhou 450001 China; ^3^ School of Medicine Nankai University Tianjin 300071 China

**Keywords:** ferritin, heterogeneous nanostructures, protein nanocages, self‐assembly, tumor

## Abstract

Well‐defined nanostructures are crucial for precisely understanding nano‐bio interactions. However, nanoparticles (NPs) fabricated through conventional synthesis approaches often lack poor controllability and reproducibility. Herein, a synthetic biology‐based strategy is introduced to fabricate uniformly reproducible protein‐based NPs, achieving precise control over heterogeneous components of the NPs. Specifically, a ferritin assembly toolbox system is developed that enables intracellular assembly of ferritin subunits/variants in *Escherichia coli*. Using this strategy, a proof‐of‐concept study is provided to explore the interplay between ligand density of NPs and their tumor targets/penetration. Various ferritin hybrid nanocages (FHn) containing human ferritin heavy chains (FH) and light chains are accurately assembled, leveraging their intrinsic binding with tumor cells and prolonged circulation time in blood, respectively. Further studies reveal that tumor cell uptake is FH density‐dependent through active binding with transferrin receptor 1, whereas in vivo tumor accumulation and tissue penetration are found to be correlated to heterogeneous assembly of FHn and vascular permeability of tumors. Densities of 3.7 FH/100 nm^2^ on the nanoparticle surface exhibit the highest degree of tumor accumulation and penetration, particularly in tumors with high permeability compared to those with low permeability. This study underscores the significance of nanoparticle heterogeneity in determining particle fate in biological systems.

## Graphical abstract

Creating heterogeneous nanoparticles with controllability and reproducibility remains challenging using conventional synthesis approaches. Herein, a synthetic biology‐based approach is introduced that enables the creation of a highly uniform and controllable living nanosystem, allowing for the tailoring of heterogeneous ferritin nanocages through spontaneous assembly. A proof‐of‐concept study illustrates the significant impact of nanoparticle heterogeneity on both tumor uptake and penetration.

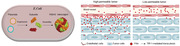

## 1 Introduction

Tumor‐targeted nanomedicines have shown promising potential to revolutionize anti‐tumor therapies.^[^ ^1^ ^]^ A notable advantage of these nanomedicines is their inherent capacity for heterogeneous functionalization. For example, the modification of anti‐tumor nanomedicines with poly(ethylene glycol) (PEG) molecules and ligands synergistically enhances tumor targeting capabilities by improving blood circulation time and actively binding to specific tumor cell receptors, respectively. Consequently, these functionalization methods have become popular design rationales in the tumor‐targeting nanomaterials community.^[^ ^2^ ^]^ However, unanswered questions persist regarding heterogeneous functionalization in nanomedicine design. Issues such as the optimal ligand number/density of nanoparticles (NPs) for tumor targeting,^[^ ^3^ ^]^ and whether this number/density differentially affects in vitro and in vivo tumor targeting need clarification numerouns NPs with tunable ligand densities have been synthesized using conventional method.^[^ ^4^ ^]^ However, a key issue in utilizing these NPs for evaluating nanoparticle‐tumor interactions is their poor controllability and reproducibility.

Modular assembly strategies offer accurate and quantitative techniques to achieve NPs with desired functionalities, but achieving precise modular assembly remains challenging when employing conventional approaches. Recently, synthetic biology‐based approaches have shown promise for the modular engineering of nanomedicines using living building materials.^[^ ^5^ ^]^ Inspired by nature and assembly of protein building blocks into exquisite nanostructures, the modular assembly of nanomedicines using well‐defined and living protein components aims to overcome the limitations of controllability and reproducibility. Ferritin, a natural protein that assembles into heterogeneous protein nanostructures consisting of 24 subunits of heavy and light ferritin chains,^[^ ^6^ ^]^ is considered an ideal platform for precisely controlling the heterogeneous functionalization of nanostructures. Although the detailed biological function of ferritin is not fully understood, genetically recombinant heavy chain ferritin nanocages (FTn) have been widely applied in tumor theranostic studies due to their intrinsic and selective affinity to transferrin receptor 1 (TfR‐1),^[^ ^7^ ^]^ which is abundantly expressed on many tumor cells. Furthermore, the inherent self‐assembly property of FTn endows them with the benefits of drug loading and multi‐functional design.^[^ ^8^ ^]^ In other words, FTn, involving 24 ferritin subunits, are easily assembled into heterogeneous structures by adjusting the stoichiometric ratio of differentially modified ferritin subunits. These heterogeneous structures are suitable for precisely understanding nano‐bio interactions, including but not limited to the interaction between the ligand number of NPs and tumor targeting. Until now, the heterogeneous assembly of FTn has merely been accomplished through an in vitro disassembly–reassembly approach, involving a multi‐step process that increases operational complexity and, consequently, potentially leads to low recovery yield and protein aggregation/instability.

**Figure 1:**
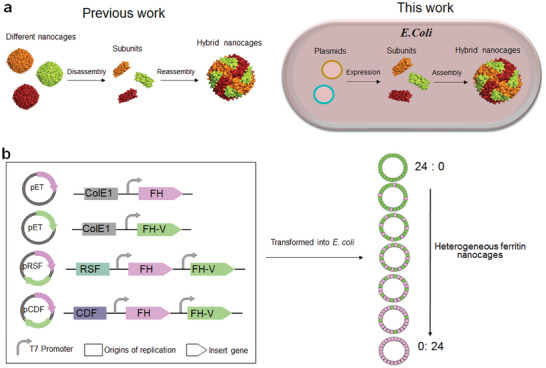
Intracellular self‐assembly strategy based on FATS. a) Schematic illustration of FHn obtained by different approaches. FHn were obtained by assembling different ferritin subunits through a sequential disassembly and reassembly procedure in vitro (left) and direct intracellular self‐assembly of ferritin heterogeneous subunits expressed in *E. coli* (right). b) Different ratios of heterogeneous ferritin nanocages were intracellularly self‐assembled based on FATS, consisting of combinations of four plasmids.

In this study, we leverage synthetic biology technology to establish a novel ferritin assembly toolbox system (FATS) for the intracellular in‐situ assembly of ferritin subunits/variants. Unlike in vitro disassembly–reassembly approach, this system allows for the direct assembly of heterogeneous FTn containing different functional components in *Escherichia coli* (*E. coli*) (**Figure** **1**a). The FATS consists of four ferritin‐based plasmid systems anchored with different open reading frames (ORF) and ferritin or its variant sequences, enabling the precise construction of heterogeneous FTn in a controlled manner. Using the FATS, we assembled five FTn‐based nanocarriers with different ligand densities from ferritin heavy chain (FH)/ferritin light chains (FL). We also assessed the effect of these varied assemblies on tumor cell uptake, their tumor accumulation dynamics, and their ability for tumor tissue penetration. In particular, we compared the in vivo tumor targeting ability of the assemblies in tumors with varying levels of vascular permeability, including both high‐ and low‐permeable tumors. Finally, we explore the in vivo anti‐tumor efficacy of the assemblies following their loading with a chemotherapeutic drug, doxorubicin (Dox).

## 2 Results

### 2.1 Design and Construction of FATS

FTn are formed by the intrinsic self‐assembly of 24 ferritin subunits, allowing for the theoretical design of various heterogeneous nanocages by accurately controlling different ingredient ratios of the 24 subunits. We herein developed a FATS to enable the direct expression of different subunits, thereby facilitating precise control over the self‐assembly of heterogeneous nanocages within *E. coli*. The FATS comprised four ferritin‐based plasmids: one with the FH sequence; one with genetic variants of the FH sequence (FH‐V); and two different co‐expression plasmids (i.e., bicistronic vectors) containing both the FH and FH‐V sequences (Figure 1b). For plasmid expression systems, the promoter propensity to drive expression and the copy numbers of open reading frame sequence tend to determine downstream the expression efficiency of the protein of interest. To better monitor protein soluble expression, the four plasmids contained the same T7 promoters but had varied ORF. Specifically, FH and FH‐V were anchored with the ColE1 replicon (ColE1 ori), while the two bicistronic vectors differed, with one containing the CDF replicon (CDF ori) and the other having the RSF replicon (RSF ori). Following the transformation of one or more plasmids into *E. coli*, our system enabled the spontaneous assembly of differentially expressed ferritin subunits/variants, allowing for the direct acquisition of homogeneous/ heterogeneous FTn‐based NPs without the need for any further modifications. In other words, the products of the FH, FH‐V and two FH/FH‐V plasmids could theoretically be co‐assembled into nanocages and the assembly ratio could be freely controlled based on its dependency on plasmid combinations.

**Figure 2:**
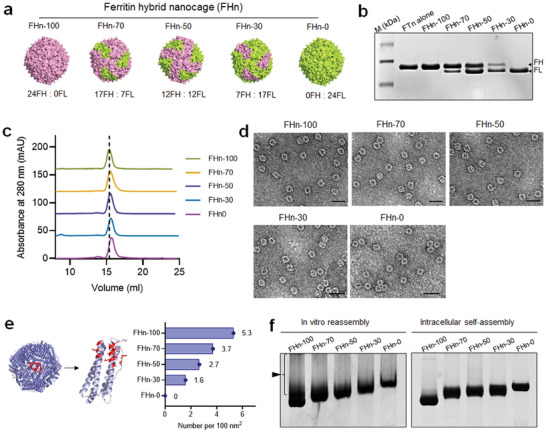
Assembly of FHn using FH and FL. a) Schematic illustration of five FHn containing different FH and FL. Various nanocages were composed of 24 subunits and the hybrid nanocages were assembled by adjusting the ratio of FH to FL via FATS. b) SDS‐PAGE gel electrophoresis analysis of five FHn engineered with different fractions of FH. c) Size exclusion chromatography analysis of the size of various FHn in protein purification equipment. d) Representative TEM images of various FHn. Scale bar = 20 nm. e) Protein structure‐based quantification analysis of FH densities on FHn surface. Red, the binding site of FH with TfR‐1. The calculation is based on the assumption of a ferritin diameter of 12 nm, resulting in an approximate surface area of 452.16 nm^2^. With each subunit featuring a single ligand binding site, the ligand density is determined by calculating the subunit number per 100 nm^2^ of surface area. f) Native‐PAGE gel electrophoresis analysis of the stability of FHn obtained by in vitro reassembly (left) and intracellular self‐assembly (right) strategies. Arrow indicates the aggregated FHn in gel.

Next, we sought to develop an application scenario based on the advantages of FATS. To achieve this, we designed a highly controllable and reproducible NP system to study the effect of heterogeneous FTn‐based NPs on tumor‐targeted drug delivery. FH contains a motif that imparts intrinsic binding affinity to TfR‐1,^[^ ^9^ ^]^ highly expressed on many tumor cells. Blood serum naturally contains ferritin composed of FL, suggesting that FL is capable of prolonged circulation in the bloodstream. Thus, we considered the co‐assembly of FH and FL (i.e., FH‐V) by taking advantage of the established FATS, to understand the effects of heterogeneous NPs on tumor uptake and penetration. Our hypothesis was that in a typical ferritin hybrid nanocage (FHn) assembled from FH and FL, FH provides precisely tunable ligand density, while FL enhances the blood circulation time of the particles in bloodstream. As illustrated in **Figures** **2**a and S1a (Supporting Information), FHn with five ligand densities (i.e., FH) were obtained by transforming *E. coli* with the plasmid alone or a different combination of plasmids. The plasmid combinations and resultant FHn were 100% FH/0% FL (i.e., 24 FH subunits, FHn‐100), 70% FH/30% FL (i.e., 17 FH/7 FL subunits, FHn‐70), 50% FH/50% FL (i.e., 12 FH/12 FL subunits, FHn‐50), 30% FH/70% FL (i.e., 7 FH/17 FL subunits, FHn‐30), and 0% FH/100% FL (i.e., 24 FL subunits, FHn‐0). This outcome was confirmed by SDS‐PAGE analysis (Figure 2b). Size exclusion chromatography analysis revealed that the sizes of the FHn gradually decreased as the proportion of FL increased (Figure 2c), as evidenced by the larger molecular weight of FH (≈21.1 kDa vs. FL ≈19.9 kDa). The hollow cavities of various FHn were clearly observable by transmission electron microscopy (TEM), exhibiting cage‐like nanostructures (Figure 2d). Subsequently, we quantified the ligand densities on the nanoparticle surfaces according to the assembled FH numbers. The surface ligand densities were determined to be 5.3, 3.7, 2.7, 1.6, and 0 ligands per 100 nm^2^ NP surface for FHn‐100, FHn‐70, FHn‐50, FHn‐30, and FHn‐0, respectively (Figure 2e). Before this study, heterogeneous assembly of FHn was mainly conducted through an in vitro disassembly‐reassembly strategy by adjusting the stoichiometric ratio of the different subunits under harsh conditions, as demonstrated in Figure 1a. To highlight the advantage of the intracellular self‐assembly strategy, we evaluated the stability of the FHn derived from the two different approaches. Native‐PAGE analysis revealed that the FHn obtained from in vitro reassembly was more easily aggregated compared to the FHn obtained from intracellular self‐assembly, as evidenced by arrow indicating decreased gel migration (Figure 2f).

### 2.2 FH Density‐Dependent Cell Uptake and Transcytosis

**Figure 3:**
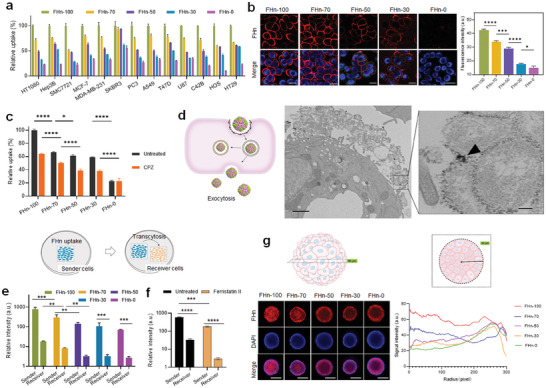
Cell uptake and penetration of FHn. a) Flow cytometry analysis of cell uptake of various FHn in different tumor cells. The uptake of FHn‐100 in each tumor cell was normalized to 100. b) Confocal images (left) and flow cytometry (right) analysis of cell binding ability of various FHn with tumor cells. Scale bar = 20 µm. *p < 0.05, ***p < 0.001, ****p < 0.0001. c) Flow cytometry analysis of cell uptake of various FHn in tumor cells without or with pre‐treatment of CPZ, an inhibitor for clathrin‐dependent endocytosis. The uptake of FHn‐100 in untreated cells was normalized to 100. *p < 0.05, ****p < 0.0001. d) Schematic illustration (left) and TEM images (right) of cell exocytosis of FHn‐100. Scale bars of TEM images are 1 µm (left) and 0.1 µm (right). Arrow indicates FHn‐100 incorporated with iron oxide NPs. e,f) Flow cytometry analysis of transcytosis mechanism by co‐incubation of sender cells and receiver cells. As shown in schematic illustration (top), the sender cells (cyan) seeded onto coverslip slides were pre‐treated with Cy5‐labeled FHn, and subsequently, the sender cells were co‐incubated with the untreated receiver cells (orange) seeded onto another coverslip slide. e) flow cytometry analysis of transcytosis ability of various FHn; **p < 0.01, ***p < 0.001. f) flow cytometry analysis of transcytosis mechanism by pre‐treatment with Ferristatin II, a small molecule to reduce TfR‐1 expression. ***p < 0.001, ****p < 0.0001. g) Distribution of the Cy5‐labeled FHn in 3D tumor spheroids. Left, representative middle section images; right, quantification analysis of the mean signal intensity in the middle section images. Radius, 0 and 300 indicate the center and edge of the tumor spheroid, respectively. Scale bar = 100 µm.

Having established the series of FHn, our next objective was to investigate the effect of ligand densities (i.e., FH) on tumor uptake both in vitro and in vivo. Given the high expression of TfR‐1 in most tumor cells, we selected thirteen tumor cell lines to assess the impact of the five FHn on cell uptake by incubating them with Cy5‐labeled FHn for 2 h. Flow cytometry analysis demonstrated that tumor cell uptake was dependent on the FH densities of FHn, with increased proportions of FH leading to elevated cell uptake (**Figure** **3**a) and cell binding (Figure S2a, Supporting Information), regardless of tumor cell type. For subsequent studies, unless otherwise stated, we chose HT29 tumor cells to explore the detailed interaction mechanisms. Cell binding assays illustrated that Cy5‐labeled FHn efficiently attached to the tumor cell surface, with prominent binding observed in the FHn‐100 and FHn‐70 groups, while the cell binding capacity of FHn‐0 was negligible (Figure 3b). Our previous research demonstrated that FH binds TfR‐1 to mediate cellular uptake through clathrin‐dependent endocytosis.^[^ ^10^ ^]^ To further understand the cellular uptake mechanisms, we pre‐treated cells with the clathrin‐dependent endocytosis inhibitor chlorpromazine (CPZ). We observed a significant inhibition effect on the cell uptake of FHn containing FH. In contrast, CPZ had no impact on the cell uptake of FHn‐0 (Figure 3c). These results indicated that tumor cell uptake of FHn was dependent on TfR‐1 binding.

TfR‐1 plays a crucial role in transcellular transport mechanisms (i.e., transcytosis), owing to its unique internalization and recycling functions, making it an attractive candidate for drug targeting and therapeutic delivery.^[^ ^11^ ^]^ We thus sought to study the effect of different FH densities on TfR‐1‐dependent transcytosis by tumor cells. To explore the intracellular fate of FHn after uptake, tumor cells were incubated with Cy5‐labeled FHn, and lysosomes were stained with LysoTracker. Confocal images and Pearson's coefficient analysis revealed that FHn with varying FH densities co‐localized with lysosomes following uptake (Figure S1b, Supporting Information). In contrast, a significant decrease in co‐localization was observed for FHn‐0, attributed to the absence of TfR‐1‐mediated uptake. We further explored whether cell exocytosis of FHn‐100 occurred after binding and uptake via TfR‐1. To visualize FHn‐100 under TEM, iron oxide NPs were generated in the hollow cavity of FHn‐100 using a biomimetic synthesis approach described in previous studies.^[^ ^10^, ^12^ ^]^ TEM images of tumor cell samples revealed a substantial presence of extracellular FHn‐100 attached to the surface of exocytotic vesicles (Figure 3d). Further study focused on whether FHn could be delivered by transcytosis. In a transcytosis assay, after the uptake of Cy5‐labeled FHn by tumor cells (i.e., sender cells), slides with sender cells were placed in dishes alongside slides seeded with untreated tumor cells (i.e., receiver cells). Flow cytometry analysis indicated a decline in signal intensity for both sender and receiver cells with lower FH densities (Figure 3e). The addition of Ferristatin II, a suppressor of TfR‐1 expression, into the transcytosis assay, resulted in an ≈11‐fold reduction in FHn‐100 uptake by Ferristatin II‐treated receiver cells compared to untreated receiver cells (Figure 3f). These findings indicated that FHn was excreted by exocytosis and subsequently internalized by adjacent tumor cells. This active transcytosis mechanism was dependent on FH densities and/or TfR‐1. Building upon the capacity for transcytosis, we explored whether FH densities facilitated deep penetration into tumor tissue using 3D tumor spheroid models established with HT29 cells. Confocal imaging of tumor spheroids treated with FHn was conducted by laser scanning from the surface through to the middle cross‐sections of the spheroids (Figure S2b, Supporting Information). For sections with a tissue depth of 94 µm, confocal images demonstrated that FHn‐100 and FHn‐70 were uniformly distributed throughout the entirety of the spheroids, including the cores. In contrast, FHn‐30 and FHn‐0 were primarily located at the periphery of the tumor spheroid (Figure 3g, left). Quantification analysis of signal intensity from the core to the surface (i.e., pixels from 0 to 300) revealed FH density‐dependent penetration, with FHn‐100 and FHn‐70 exhibiting significantly stronger penetration abilities compared to FHn‐50, FHn‐30, and FHn‐0 (Figure 3g, right).

### 2.3 Vascular Permeability and Tumor Tissue Penetration

We next sought to explore the in vivo behaviors of i.v. administered FHn in mice bearing subcutaneous tumors. After administration, blood circulation, vascular transport, and tissue penetration are crucial prerequisites for the targeted delivery of nanomedicines to tumor cells. First, we compared the blood pharmacokinetics of FHn. As expected, FHn‐0 alone exhibited optimal blood pharmacokinetics, and the incorporation of FL provided a prolonged blood circulation time for FHn. Compared to FHn‐100 and FHn‐70, FHn‐0 demonstrated substantially improved blood pharmacokinetics with 2.5‐ and 2.0‐fold greater area under a curve (AUC), respectively (Figure S3, Supporting Information). Next, we sought to study the vascular transport of various FHn using our recently developed a machine learning (ML)‐based single‐vessel analysis method. The workflow of the method, as depicted in Figure S4a (Supporting Information), consists of multiple steps. ML‐based automatic segmentation of input images enables the quantitative assessment of FHn vascular permeability in each vessel by determining vessel coverage area and FHn penetration area. Analysis of randomly established tumor models revealed that HT29 and SKBR3 had the highest and lowest permeabilities for FHn‐100, respectively (Figure S4b, Supporting Information). Vascular penetration ability of NPs in tumors plays a substantial role in achieving effective therapeutic delivery outcomes, and thus, HT29‐ and SKBR3‐derived tumors were chosen as representative high‐ and low‐permeable tumors for the subsequent studies, respectively. By comparing the effect of FH densities on tumor vascular permeability, we observed that the mean permeable area (Ap) of different FHn were comparable at 0.5 h following administration, regardless of high‐permeable (Figure S4c, Supporting Information) and low‐permeable tumors (Figure S4d, Supporting Information).

**Figure 4:**
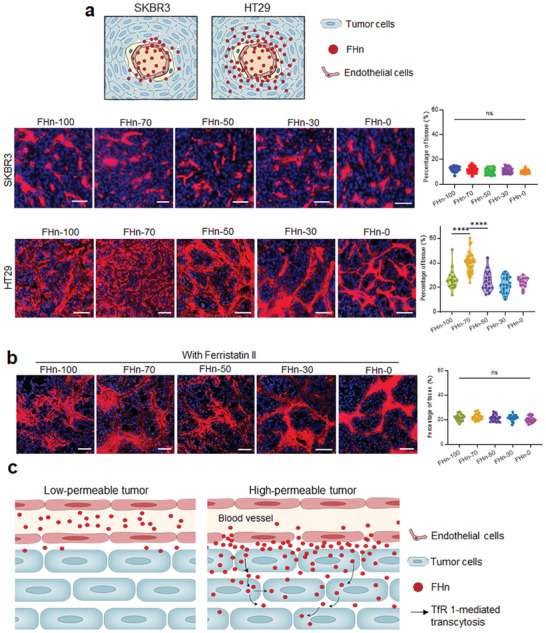
In vivo tumor tissue penetration. a) Tissue distribution of various FHn in SKBR3 and HT29 tumors following systemic administration. Representative confocal images (left) and image‐based quantification analysis (right) of Cy5‐labeled FHn distribution in low‐ and high‐permeable tumor tissues (n = 3 per group). Quantification analysis was performed by determining the coverage area of various FHn in tumor tissues. Scale bar = 100 µm. ****p < 0.0001. b) Tissue distribution and quantification analysis of various FHn in HT29 tumors pre‐treated with Ferristatin II (n = 3 per group). Scale bar = 50 µm. c) Diagram illustrating the dominant tissue penetration mechanism in low‐permeable tumors and high‐permeable tumors. A large amount of the FHn in blood circulation easily passes through tumor blood vessels in high‐permeable vasculatures based on a passive extravasation mechanism. Then, TfR‐1‐mediated active transcytosis mechanism facilitated deep penetration of FHn into solid tumor tissue. In contrast, the limited extravasated FHn beyond blood vessels in low‐permeable tumors greatly reduced their tissue penetration capacity.

Next, we compared the tumor tissue penetration of different FHn in two types of permeable tumors following systemic administration. Confocal images demonstrated that the FHn could penetrate deeply into the tumor tissue across HT29 tumor vasculature, especially evident in the FHn‐100, FHn‐70, and FHn‐50 groups (**Figure** **4**a). In contrast, tissue penetration of all FHn was limited in SKBR3 tumors. Quantification of the percentage of particle distribution area in whole tumor tissues indicated that the distribution of FHn in HT29 tumor tissue was at least two‐fold higher than that in SKBR3 tumor tissue under identical conditions (Figure S5a, Supporting Information). Importantly, we did not observe significant differences in FHn distribution in SKBR3, but the distribution of FHn‐70 in HT29 tumor tissue was 1.50‐, 1.58‐, 1.81‐, and 1.64‐fold greater than FHn‐100, FHn‐50, FHn‐30 and FHn‐0, respectively. To further study the penetration mechanism of FHn, we explored whether TfR‐1‐mediated transcytosis played a key role in the tissue penetration process. First, we evaluated the expression level of TfR1 in HT29 and SKBR3 cells. The results from Western blot analysis indicated a significantly higher TfR1 expression in HT29 compared to SKBR3 (Figure S5b, Supporting Information). Flow cytometry analysis also demonstrated that TfR 1 expression on the cell membrane was approximately twofold higher in HT29 than in SKBR3 (Figure S5c, Supporting Information). Additionally, inhibition of TfR‐1 expression resulted in reduced cell uptake of FHn‐100 in Ferristatin II pre‐treated tumor cells (Figure S5d, Supporting Information). In contrast, Ferristatin II had no apparent change in FHn‐0 tumor cell uptake. Subsequently, the tissue penetration of various FHn in HT29 tumor tissues was assessed following localized pre‐treatment with Ferristatin II. Confocal images and image‐based quantification analysis demonstrated that the tissue penetration of various FHn was comparable in HT29 tumors after Ferristatin II pre‐treatment (Figure 4b). Notably, Ferristatin II pre‐treatment substantially decreased tissue penetration of FHn, especially for FHn‐70 (Figure S5e, Supporting Information). Based on these results, we proposed the potential mechanisms of FHn penetration into different tumors (Figure 4c). For tumors with high permeability, higher vascular permeability resulted in a greater number of FHn being transported across blood vessels and entering the extravascular space, which in turn resulted in TfR‐1‐mediated transcytosis and facilitated deep penetration into tumor tissues due to subsequent active binding with TfR‐1. During this process, the dynamics of both blood circulation time and cell binding ability were determinants of tissue penetration of FHn, explaining the highest tissue distribution observed in FHn‐70 groups. In contrast, in low‐permeable tumors, the diminished quantity of FHn failed to adequately facilitate deep penetration within tumors due to limited FHn transport across blood vessels, thereby explaining the undifferentiated distribution throughout tumor tissue among the different FHn.

### 2.4 In Vivo Tumor Accumulation

**Figure 5:**
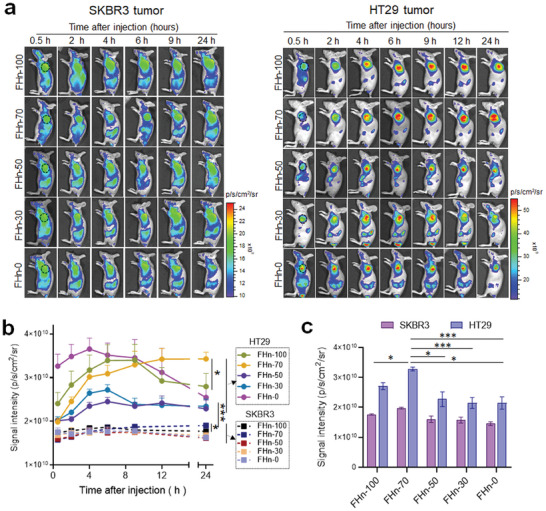
In vivo tumor accumulation. a) In vivo imaging of accumulation of systemically administrated FHn in SKBR3 (left) and HT29 tumors (right)) at the designated time points using IVIS imaging system (n = 5 per group). The inoculated tumors were indicated by black dashed circles. b) Quantification analysis of tumor uptake of various FHn according to the images from a). *p < 0.05, ***p < 0.001. c) Quantification analysis of mean signal intensity of various FHn in isolated SKBR3 and HT29 tumors from the sacrificed mice at 24 h post‐injection. *p < 0.05, ***p < 0.001.

Next, we investigated the time‐dependent in vivo tumor accumulation through fluorescent imaging of systemically administrated Cy5.5‐labeled FHn. The effect of various FHn on tumor accumulation ability was compared in both low‐permeable SKBR3 tumors and high‐permeable HT29 tumors (**Figure** **5**a,b). In high‐permeable HT29 tumor models, a gradual enhancement of tumor accumulation for FHn‐100 was observed over time, reaching maximal uptake at 6–8 h. Subsequently, the signal intensity of FHn‐100 in tumors decreased. In contrast, FHn‐0 exhibited faster tumor uptake following administration, but its accumulation rapidly declined after 6 h, significantly lower than that of FHn‐100 at 24 h. These in vivo tumor uptake dynamics were explained by the synergistic actions of tumor tissue metabolism, tumor cell targeting of FHn‐100, and the longer blood circulation time of FHn‐0. Compared to other FHn, FHn‐70 showed the highest tumor uptake at 12 h after administration. Quantification analysis of tumors isolated 24 h post‐injection further confirmed this result (Figure 5c). Similar tumor accumulation dynamics of various FHn were observed in low‐permeable SKBR3 tumors. However, the signal intensity of the five FHn in SKBR3 tumors was significantly lower than that observed in HT29 tumors at all designated time points, revealing that vascular permeability was positively correlated with the tumor uptake capabilities of FHn. Importantly, the variation in the extent of tumor uptake of FHn in SKBR3 tumors over time was evidently less than that of HT29 tumors. Additionally, quantification analysis of the signal intensity ratio of 24 to 0.5 h demonstrated that FHn‐70 had the highest tumor accumulation capability in both high‐permeable and low‐permeable tumors. The ratio of FHn‐70 in HT29 tumors was ≈1.4‐fold greater than that in SKBR3 tumors. These results demonstrated that the effect of FH densities on in vivo tumor accumulation varied in different permeable tumors, but tumor uptake of FHn‐70 was the highest amongst the various FHn, regardless of high‐ and low‐permeable tumors.

### 2.5 In Vivo Anti‐Tumor Efficacy of Dox Loaded FHn

**Figure 6:**
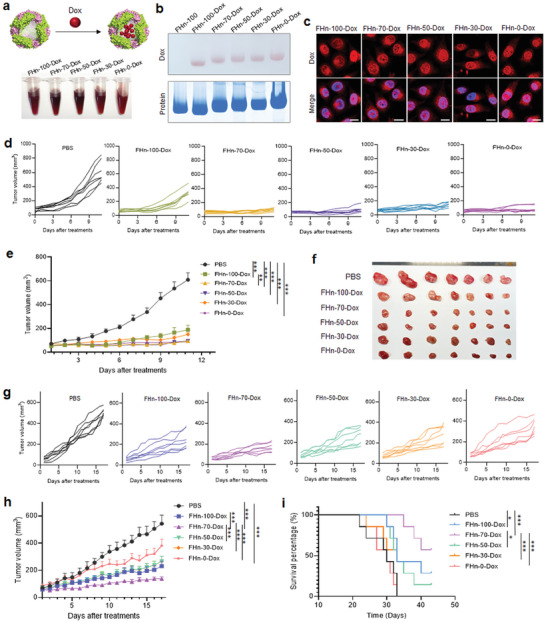
In vivo anti‐tumor ability of various FHn‐Dox. a) Dox loading into various FHn. b) Native‐PAGE gel electrophoresis analysis of various FHn‐Dox. The color of Dox was directly observed in gel (top), and the FHn was stained by coomassie brilliant blue (bottom). c) Co‐localization of Dox loaded FTn with nucleus after tumor cell uptake for 4 h. Scale bar = 10 µm. d) Tumor growth curves of each SKBR3 tumor over time following different treatments. e) Tumor growth curves of different groups of SKBR3 tumor‐bearing mice after administration of the indicated treatments (n = 7 per group). ***p < 0.001. f) The isolated SKBR3 tumors from the sacrificed mice following treatments with various FHn‐Dox. g) Tumor growth curves of each HT29 tumor over time following different treatments. h) Tumor growth curves of different groups of HT29 tumor‐bearing mice after various treatments indicated (n = 7 per group). ***p < 0.001. i) Kaplan–Meier survival curve of HT29‐bearing mice following different treatments (n = 7 per group). *p < 0.05, ***p < 0.001.

We next sought to evaluate the in vivo anti‐tumor efficacy of FHn by loading a chemotherapeutic drug, Dox.^[^ ^13^ ^]^ The inherent drug entry channel within the FHn protein shell^[^ ^14^ ^]^ allows for the direct loading of small molecular drugs into the inner cavity (**Figure** **6**a). The numbers of Dox molecules loaded into each FHn‐100 (FHn‐100‐Dox), FHn‐70 (FHn‐70‐Dox), FHn‐50 (FHn‐50‐Dox), FHn‐30 (FHn‐30‐Dox) and FHn‐0 (FHn‐0‐Dox) were determined to be 37 ± 2, 29 ± 1, 26 ± 2, 30 ± 2, and 21 ± 2, respectively. Native‐PAGE gel electrophoresis analysis demonstrated successful loading of Dox into the FHn, as evidenced by the co‐localization of Dox and protein (Figure 6b). A crucial target for the cytotoxic actions of Dox is the nucleus, where Dox intercalates with DNA to form DNA adducts.^[^ ^15^ ^]^ To verify the intracellular distribution of Dox, tumor cells were incubated with FHn without or with Dox for 4 h and confocal microscopy was used to visualize Dox, predominantly located in nuclei (Figure 6c). FHn primarily co‐localized with lysosomes, as demonstrated in Figure S1b (Supporting Information), suggesting that Dox was successfully released after uptake and entered the nuclei of tumor cells. All five of the FHn‐Dox also exhibited a concentration‐dependent inhibition effect on HT29 cells (Figure S6, Supporting Information).

The impact of different Dox‐loaded FHn on tumor growth was assessed through systemic administration every 3 days in mice harboring xenografts of low‐permeable SKBR3 tumors and high‐permeable HT29 tumors. Treatment with various Dox‐loaded FHn displayed significant efficacy in controlling SKBR3 tumor growth compared to PBS treatment alone (Figure 6d,e). The results were further confirmed by isolating tumors following three doses of Dox‐loaded FHn (Figure 6f). However, we did not observe significantly distinct suppressive effects among the various Dox‐loaded FHn in SKBR3 tumors. For the high‐permeable HT29 tumors, treatment with various Dox‐loaded FHn exhibited significantly varied inhibited effects on tumor growth (Figure 6g,h). Importantly, FHn‐70‐Dox demonstrated a superior suppressive effect on tumor growth compared to other Dox‐loaded FHn. Survival rates were also enhanced for mice treated with FHn‐100‐Dox, FHn‐70‐Dox, FHn‐50‐Dox, and FHn‐30‐Dox, compared to PBS‐treated mice. The median survival for mice treated with FHn‐100‐Dox, FHn‐50‐Dox, and FHn‐30‐Dox was 33, 33, and 31 days, respectively (Figure 6i). In contrast, treatment with FHn‐70‐Dox resulted in a 57% progression‐free survival after 43 days. Of note, in comparison to HT29, identical Dox‐loaded FHn exhibited a more pronounced inhibitory effect on SKBR3 tumor growth, primarily attributed to their increased sensitivity to Dox (Figure S7a, Supporting Information). Additionally, no apparent systemic toxicity of the Dox‐loaded FTn‐based NPs was observed, as evidenced by evaluation of body weight changes in the mice and histological analysis of major organs (Figure S7b,c, Supporting Information). Collectively, these results suggest that by modulating FH densities incorporated into FHn, it is valuable to design FTn‐based NPs for the targeted treatment of high‐permeable tumors. However, FH densities did not appear to play important roles in mitigating low‐permeable tumors due to limited vascular permeability and subsequent tissue penetration.

## 3 Discussion

In this study, we constructed a FATS by taking advantage of a synthetic biology strategy, enabling precise and quantitative assembly of highly ordered heterogeneous protein nanostructures. Using this technology, we successfully assembled various FHn by adjusting the proportions of moieties (i.e., FH and FL), allowing for tunable protein motifs to influence tumor cell binding and blood circulation time. FHn yielded through FATS provided uniform and reproducible model NPs, offering tools to understand the intricate relationship between NPs and tumor uptake/penetration. Our findings revealed a positive correlation between tumor cell uptake and the density of FH, irrespective of tumor cell type. In contrast, achieving optimal in vivo tumor accumulation/penetration among the prepared FHn required a delicate balance between FH density and blood circulation time. Consequently, FHn‐70 (i.e., 3.7 ligands per 100 nm^2^) emerged as an optimal nanocarrier, exhibiting superior tumor accumulation/penetration compared to other FHn generated in this investigation. Furthermore, our results underscore the potential for modulating the tumor accumulation and tissue penetration of NPs through rational design, particularly effective in the targeted treatment of high‐permeable tumors. In this regard, FHn was specifically designed to elucidate the interplay between nanoparticle FH density and tumor accumulation/ penetration. Future studies could expand upon these findings to determine whether optimal ligand densities elicit specific outcomes when engineering other conventional nanosystems.

Mammalian ferritins naturally consist of two types of subunits, namely FH (21 kDa) and FL (19 kDa). These two subunits co‐assemble in various proportions to form a 24‐subunit protein nanocage with tissue‐specific distribution.^[^ ^16^ ^]^ Although the detailed co‐assembly mechanism is not fully understood, the assembly of FH and FL may begin with the formation of homodimer of ferritin subunits.^[^ ^17^ ^]^ The subsequent stepwise assembly into octahedral symmetry nanocages involves three fourfold symmetry axes, eight threefold axes, and six twofold axes through inter‐ and intra‐subunits interactions. These nanocages have been extensively utilized as nanocarriers for drug delivery, using either genetically recombinant FH alone or FL alone.^[^ ^18^ ^]^ However, reports on the hetero‐assembly of FH and FL for drug delivery are scarce. Herein, we present a synthetic biology‐based strategy for the preparation of heterogeneous ferritin nanocages by precisely controlling the FH to FL ratio. This approach involves the transformation of one or more plasmids into *E. coli*, enabling the modulation of FH and/or FL soluble expression by adjusting promoter propensity and copy numbers. As these ingredients assemble into a 24‐subunit nanocage, higher expression levels of FH or FL indicate an increased likelihood of both inter‐ and intra‐subunit interactions. Consequently, the soluble expression levels of FH and/or FL directly correspond to the proportions of each component in the assembled nanocages. This assembly structure allows for a varied proportion of the two ingredients within a fixed total number. Going forward, as demonstrated in this study, we anticipate that synthetic biology‐based strategies will lead the way in the rational design of next‐generation anticancer nanomedicines.

Poor tumor tissue penetration is a major challenge and bottleneck for NPs in achieving efficacious anticancer therapy.^[^ ^19^ ^]^ TfR‐1, with its distinctive sorting and recycling functions, has emerged as an attractive receptor for orchestrating targeted drug delivery, especially in scenarios involving transcytosis‐mediated delivery.^[^ ^20^ ^]^ This study highlights the pivotal role played by TfR‐1‐mediated transcytosis in facilitating profound NP penetration into solid tumor tissue. In the transcytosis process detailed here, a substantial quantity of FHn actively entered endosomes through TfR‐1 binding and clathrin‐mediated uptake. Following this internalization, a fraction of FHn was excreted into the extracellular space through exocytosis, guided by TfR‐1‐mediated receptor sorting and recycling to the cell surface. Subsequently, neighboring tumor cells internalized these exocytotic FHn, leading to a gradual penetration of NPs into the deeper tumor tissue. This sequential cascade event resulted in a gradual dilution of NPs from the abundance periphery of blood vessels to lower concentrations in the deeper layers of the tumor tissue. Thus, the efficacy of the initial step (i.e., vascular permeability) emerged as a crucial determinant for NP penetration into tumor tissues. Additionally, the presence of high interstitial fluid pressure within tumor tissue further limits the deep penetration of NPs relying solely on passive diffusion mechanisms.^[^ ^21^ ^]^ Active transcytosis mediated by receptor, such as TfR‐1, holds promising potential for enhancing NP penetration into tumor tissues. Beyond this study, several potential aspects of receptor‐mediated transcytosis require further investigation, including i) understanding the relationship among receptor‐ligand affinity, ligand density on the surface of NPs, and the transcytosis efficacy; ii) investigating the interplay and adaptation between receptor distribution on tumor cell surface and ligand density on NP surfaces; iii) assessing the impact of NP features (e.g., ligands) on their distribution across various cell layers, from blood vessel cells to deep‐seated tumor tissue cells; and iv) delineating the proportional contribution of passive penetration versus receptor‐mediated active transcytosis in determining NP penetration into tumor tissue.

## 4 Experimental Section

### Plasmid Construction and Protein Purification

The construction of the ferritin assembly toolbox system (FATS) necessitates the use of expression plasmids and specific genes. In a standard FATS setup, three prokaryotic expression vectors were employed, namely pET21a, and two bicistronic vectors (pCDFDuet and pRSFDuet). These bicistronic vectors, utilizing CDF replicon (CDF ori) and RSF replicon (RSF ori), enable the co‐expression of two genes. For this study, three genes of interest were essential: human ferritin heavy chain (FH), human ferritin light chain (FL), and human ferritin heavy chain with genetic variants (FH‐V). FH and FL genes were obtained through polymerase chain reaction (PCR) amplification using cDNA from human HT29 tumor cells. Subsequently, these genes were inserted into the respective expression vectors. The pET21a vectors accommodated one gene of interest each, while pCDFDuet and pRSFDuet vectors allowed for the incorporation of different combinations of these genes. Accordingly, FH, FL, and FH‐V genes were individually inserted into pET21a vectors, while the pCDFDuet and pRSFDuet bicistronic vectors housed combinations of FH, FL, and FH‐V genes. Detailed information on plasmid construction can be found in Table S1 (Supporting Information). The accuracy of all constructions was confirmed through DNA sequencing analysis.

Ferritin nanocages were assembled through the intrinsic self‐assembly of 24 ferritin subunits, allowing for the design of heterogeneous ferritin nanocages by adjusting the ratios of different ferritin subunits. To precisely regulate the ratio of various subunits/variants of ferritin intracellularly, the previously mentioned constructions were transformed or co‐transformed into *E. coli BL21(DE3)* as required. Following transformation, the ferritin‐based subunits expressed in each *E. coli* strain facilitated self‐assembly into a single nanocage intracellularly. Throughout this process, the ratios of different ferritin subunits were precisely controlled by adjusting the expression levels of target subunits in *E. coli*. For instance, the hybrid nanocages with 12 FH and 12 FL were produced from *E. coli*‐A. To enhance the FH ratio, the pET21a vector anchored with FH was additionally transformed into *E. coli*‐A. Conversely, the pET21a vector anchored with FL was introduced to reduce the FH ratio in each hybrid nanocage. The strategies employed for transforming different heterogeneous nanocages are outlined in Table S2 (Supporting Information). The resulting heterogeneous nanocages underwent verification of the ratios of different subunits through SDS‐PAGE analysis and size exclusion chromatography analysis.

Following plasmid transformation into *E. coli*, various FHn were generated through protein expression and purification, following established protocols.^[^ ^22^ ^]^ Briefly, the engineered *E. coli* were cultivated in Luria–Bertani medium at 37 °C. protein expression was induced by 1 mm isopropyl β‐D‐thiogalactoside (IPTG) when the cells reached an absorbance at 600 nm of 0.6. The additional 4 h of culturing at 37 °C allowed protein expression and self‐assembly. After cell collection and sonication, the soluble fraction was heat‐treated at 60 °C for 15 min and then centrifuged at 12 000 g for 10 min. The supernatant was collected and concentrated using an Amicon‐Ultra (30 kDa, Millipore). Subsequent purification was carried out using the AKTA pure protein purification system (Cytiva) equipped with a Superose 6 increase column (GE Healthcare). The resulting diverse nanocages were visualized by transmission electron microscopy (TEM, HITACHI HT7700) via negative staining with 1% Uranyl Acetate. Molecular weights and sizes of the nanocages were determined by SDS‐PAGE and size‐exclusion chromatography. Protein structure analysis was conducted using PyMOL v.2.3.2 based on PDB ID 3AJO.

### In Vitro Disassembly‐Reassembly of Nanocages

For the in vitro reassembly of FHn, the two‐step disassembly/reassembly procedures were performed by adjusting buffer pH. Briefly, the FHn in PBS (pH 7.4) at an overall protein concentration of 0.4 µm was disassembled by adjusting the pH to 2 for 20 min incubation. Then the pH was adjusted back to 7.0–7.4, followed by another 6 h incubation for reassembly. The resulting samples were concentrated and analyzed by SDS‐PAGE analysis.

### Labeling and Dox Loading of Different FHn

For labeling of fluorescence dyes, various FHn were reacted with 30 molar equivalents of Cy5‐NHS ester or Cy5.5‐NHS ester (Lumiprobe) at 4 °C for 12 h in PBS solution (pH 8.0). The resulting mixtures were purified with a PD‐10 desalting column (GE Healthcare). The dye numbers for each FHn were determined to be in the range of 6–8.

To load Doxorubicin (Dox) into FHn (FHn‐Dox), FHn stock solution diluted in 20 mm Tris‐HCl buffer (pH 8.0, containing 100 mm NaCl and 10% glycerin) to a final concentration of 1 mg mL^−1^. After incubation in 60 °C water bath for 10 min, Dox was added to reach the final concentration of 0.3 mg mL^−1^. The mixture was then continuously stirred at 60 °C for 4 h protected from light. After Dox loading, the resulting FHn‐Dox were concentrated by Amicon‐Ultra (30 kDa, Millipore), and purified using a PD‐10 Desalting Column (Cytiva). The successful Dox loading into FHn was confirmed by Native‐PAGE gel electrophoresis. The Dox concentration was determined by UV‐vis spectroscopy at 485 nm. The bicinchoninic acid (BCA) Protein Assay Kit (YEASEN) was used to determine the nanocage protein concentration. The loading efficiency (per nanocage) was calculated as following formula: loading efficiency (per nanocage) = [Dox concentration/molecular weight of Dox]/[protein concentration/molecular weight of nanocages].

### Interaction of Different FHn with Tumor Cells

For cell binding assays, confocal microscopy was used to determine the binding ability of various FHn to tumor cells. Specifically, HT29 cells were seeded on glass‐bottom cell culture dishes, and incubated with various FHn at 4 °C for 2 h. After fixation with 4% paraformaldehyde, the cells were stained with DAPI for 10 min. Sample observation was performed using confocal laser scanning microscopy (CLSM, LSM710, Zeiss). Flow cytometry was performed for quantitative evaluation of different FHn cell binding ability to tumor cells. Briefly, HT29 cells were resuspended in binding buffer (i.e., 2% BSA in PBS) with a concentration of 1 × 10^6^ cells mL^−1^. Then the cell suspension was incubated with different concentrations of various Cy5‐labeled (Ex/Em = 651/670 nm) FHn (6 µm) on ice for 30 min, followed by cold PBS washing. Cell binding analysis was subsequently performed using flow cytometry (BD FACSCalibur).

To study cell uptake of particles, different types of tumor cell lines, human fibrosarcoma (HT1080), human hepatocarcinoma (Hep3B), human hepatocarcinoma (SMMC7721), human breast adenocarcinoma (MCF7), human breast adenocarcinoma (MDA‐MB‐231), human breast carcinoma (SKBR3), human prostate adenocarcinoma (PC3), human lung adenocarcinoma(A549), human ductal carcinoma of the breast (T47D), human glioblastoma (U87), human prostatic carcinoma (C42B), human osteosarcoma (HOS), or human colorectal adenocarcinoma (HT29) cells, were cultured under standard cell culture conditions. After reaching the desired confluency, different Cy5‐labeled FHn (2 µm) were incubated with cells for at 37 °C in serum‐free medium. After 2 h uptake, the cells were collected for quantitative analysis via flow cytometry. To investigate the cellular uptake mechanism of various FHn, the cells were pre‐treated with Chlorpromazine (CPZ) (10 µg mL^−1^) for 1 h prior to the addition of FHn.

For assessment of subcellular localization, HT29 tumor cells were incubated with various Cy5‐labeled FHn (2 µm) for 2 h. Then the cells were incubated with 50 nm Lyso‐Tracker Red (Beyotime) for 20 min, followed by Hoechst 33342 (Beyotime) 30 min at 37 °C. Finally, the images were collected using a confocal microscope and analyzed using the Just Another Colocalization Plugin (JACoP) of ImageJ software (NIH). To observe subcellular distribution of ferritin nanocages, the nanocages needed to be visualized under TEM images. As such, iron oxide NPs were in situ incorporated into the cavity of ferritin nanocages (FTn‐IO) by a biomimetic synthesis method following the previous report.^[^ ^10^ ^]^ Subsequently, HT29 cells were treated with FTn‐IO (100 µg mL^−1^) for 6 h in serum‐free medium. The cells were fixed at 4 °C with 2.5% glutaraldehyde fixative solution overnight. The fixed cell pellets were further processed by a standard procedure for the preparation of biospecimens for TEM. The resulting samples were observed under TEM.

To study the transcytosis of different FHn, HT29 tumor cells were plated on sterile coverslips in cell culture dishes. After uptake of various FHn (4 µm) for 4 h, HT29 tumor cells which were seeded on coverslip (sender cells) were washed with PBS at least three times and then co‐cultured with coverslip pre‐seeded with untreated HT29 cells (receiver cells). The two‐cell seeded coverslips were placed in an adjacent configuration within a culture dish containing fresh medium and incubated for an additional 12 h. Finally, the cells of these two coverslips were separately collected for flow cytometry analysis.

For co‐localization of FHn‐Dox with nucleus, HT29 cells were incubated with 5 µm FHn‐Dox in serum‐free medium for 2 h. After washing with PBS, the cells were further cultured for another 4 h in fresh medium. After staining with DAPI (Southern Biotech), the images were observed with a confocal microscope.

### Inhibition of TfR 1 Expression by Ferristatin II

Ferristatin II was used to inhibit TfR1 expression of tumor cells. Briefly, HT29 cells were pre‐treated with Ferristatin II (50 µm) for 48 h. After harvesting, the cells were stained with TfR1 antibody at 4 °C for 1 h (555534, BD Biosciences), then incubated with Alexa Fluor 633‐conjugated secondary antibody (Ex/Em = 622/640 nm). Finally, the TfR1 expression on cell membrane was analyzed by flow cytometry.

### 3D Tumor Spheroid Models

To analyze tumor penetration of different FHn, 3D tumor spheroids were prepared by modifying a previously reported protocol.^[^ ^8^ ^]^ Briefly, 1.5 × 10^5^ HT29 suspended in 3% matrigel were seeded in 96‐well microplates pre‐coated with 1% agarose. The plates were centrifuged at 500 g for 10 min, and cultured for 5–7 d. When the diameter of tumor spheroid reached 200 µm, 4 µm of various FHn were added and cultured for another 12 h. The fluorescence image was captured using confocal microscopy in a Z‐Stack module with 24 µm intervals. The acquired images were analyzed using the ImageJ software.

### Animal Studies

All animal studies were handled in accordance with the policies and guidelines of the Animal Ethics Committee of Nankai University (2022‐SYDWLL‐000189).

For plasma pharmacokinetic studies, various Cy5‐labeled FHn were administrated into Kunming mice via the tail vein at a dose of ≈20 mg kg^−1^ (protein amount with equivalent Cy5, n = 3 per group). The blood samples from tail vein were collected at the designed time points, and then the serum fluorescence intensity of Cy5 was measured using a multimode microplate reader (ANSI/SBS 3–2004).

To study the biodistribution of FHn, BALB/c nude mice bearing HT29 or SKBR3 tumor (n = 5 per group) were intravenously injected with various Cy5.5‐labeled (Ex/Em = 683/703 nm) FHn (20 mg kg^−1^). Subsequently, tumor accumulation profiles were monitored on the front flank tumors using IVIS Spectrum imaging system (Xenogen) at designed time points. After 24 h, the animals were euthanized to harvest the tumors for ex vivo imaging via the IVIS system. The fluorescence images of tumors were quantified by measuring signal intensity at the region of interest using the IVIS imaging system and accompanying software.

To evaluate tumor vascular permeability, HT29, SMMC7721, MCF7, U87, and SKBR3 tumor models were established in BALB/c nude mice (n = 3–4 for each tumor type). Once the tumor volume reached 200 mm^3^, 200 µL of Cy5‐labeled FTn (20 mg kg^−1^) were intravenously injected into mice via tail vein. Following 30 min, tumors were collected for cryosection at a thickness of 9 µm, followed by immunofluorescence staining of blood vessels using PE‐labeled anti‐CD31 antibody (MEC13.3, Biolegend). Then the samples were mounted with DAPI and were further observed under confocal microscopy, to determine the signal of DPAI labeled nucleus (Ex/Em = 405/461 nm), CD31‐labeled vessels (Ex/Em = 565/578 nm), and Cy5‐labeled FTn (Ex/Em = 633/670 nm). To better quantify vascular permeability, the image acquisition parameters for confocal microscopy were kept constant in different cryosections of various tumors. To quantitatively analyze single‐vessel permeability of various FHn in different tumor types, the acquired confocal images are automatically segmented using machine learning‐based method (i.e., Nano‐ISML) established in the recent work^[^ ^23^ ^]^ with some modifications. The training model utilized for automatic segmentation of images was consistent with the Nano‐ISML. The feature extraction and data mining were along with minor modifications. Briefly, after feature extraction of the images automatically, two features including the total FTn coverage area for each vessel (A_FTn_) and the coverage area of each vessel (Agreen), were analyzed. The vascular permeability (A_P_) is calculated by the following formula: A_P_ = A_FTn_ − A_vessel_.

For evaluation of tumor tissue penetration, HT29 and SKBR3 tumor models were injected with 200 µL of Cy5‐labeled FHn (≈20 mg protein kg^−1^) via the tail vein, respectively (n = 3 per group). To compare tissue penetration ability, various FHn with equivalent Cy5 were administrated into mice. After 2 h, the mice were sacrificed for tumor tissue collection. Cryosections were prepared with a thickness of 9 µm and then stained with DAPI. The resulting samples were observed using confocal microscope. To investigate whether the penetration of FHn in HT29 xenograft model was TfR1‐dependent, Ferristatin II (50 µm, 50 µL) was intratumorally injected to pre‐treatment for 48 h. Prior to animal euthanasia, the mice were injected with Cy5‐labeled FHn via tail vein. Following acquiring confocal images, the coverage area of Cy5 signal in tumor tissue was quantified with ImageJ software, to determine tissue penetration capacity of FHn.

For in vivo antitumor activity, female BALB/c‐nude mice were subcutaneously injected with HT29 and SKBR3, respectively. After the tumor growth, the mice xenografted with HT29 or SKBR3 tumor models were randomly divided into six groups (n = 7 per group). The mice were intravenously administered with PBS, FHn‐100‐Dox, FHn‐70‐Dox, FHn‐50‐Dox, FHn‐30‐Dox, and FHn‐0‐Dox at 3 mg kg^−1^ (body weight) Dox equivalent every 3 days. The tumor growth and the body weight were monitored every day, and the tumor volume was calculated as follows: (tumor length) × (tumor width)^2^/2. For tumor survival, the mice were monitored daily and considered dead when the tumor volume reached 1500 mm^3^.

### Statistical Analysis

All data graphs were generated with GraphPad Prism 9 and differences between groups were assessed by students *t*‐test or One‐way ANOVA. The differences in survival were analyzed using the log‐rank test. *p* < 0.05 was considered statistically significant. Results were shown as the mean ± standard error of the mean (SEM).

## Conflict of Interest

The authors declare no conflict of interest.

## Author Contributions

Q.L. collected data, conducted data analysis, and performed all experiments. C.W., M.Z., and Q.D. assisted protein preparation and animal studies. J.L. assisted in the studies of 3D tumor spheroids. A.C.M. edited the manuscript and gave data visualization advice. R.L., B.J., D.K., and Q.C. helped to guide part of experiments. X.H. and J.Z. designed and supervised all studies and wrote the manuscript. All authors have given approval to the final version of the manuscript.

## Supporting information

Supporting Information

## Data Availability

The data that support the findings of this study are available from the corresponding author upon reasonable request.;
