# Supplementary material for: Self‐Assembly of Heterogeneous Ferritin Nanocages for Tumor Uptake and Penetration
Source: Adv Sci (Weinh). 2024 Feb 17;11(17):2309271. doi: 10.1002/advs.202309271 (PMC11077646; doi:10.1002/advs.202309271)
Supplement: Supplementary file 1 — Supporting Information [file ADVS-11-2309271-s001.pdf]

ADVANCED SCIENCE Open Access

Supporting Information

for *Adv. Sci.*, DOI 10.1002/advs.202309271

Self-Assembly of Heterogeneous Ferritin Nanocages for Tumor Uptake and Penetration

Qiqi Liu, Chunyu Wang, Mingsheng Zhu, Jinming Liu, Qiannan Duan, Adam C. Midgley, Ruming Liu, Bing Jiang, Deling Kong, Quan Chen, Jie Zhuang and Xinglu Huang**

Supporting Information

Self-assembly of Heterogeneous Ferritin Nanocages for Tumor Uptake and Penetration

Qiqi Liu[a], Chunyu Wang[a], Mingsheng Zhu[a], Jinming Liu[a], Qiannan Duan[a], Adam C. Midgley[a], Ruming Liu[a], Bing Jiang[b], Deling Kong[a], Quan Chen[a], Jie Zhuang[c*], Xinglu Huang[a*]

[a]State Key Laboratory of Medicinal Chemical Biology, Key Laboratory of Bioactive Materials for the Ministry of Education, College of Life Sciences, and Frontier of Science Center for Cell Response, Nankai University, Tianjin 300071, China.

[b]Nanozyme Medical Center, School of Basic Medical Sciences, Zhengzhou University, Zhengzhou 450001, China

[c]School of Medicine, Nankai University, Tianjin 300071, China.

* Email: zhuangj@nankai.edu.cn; huangxinglu@nankai.edu.cn

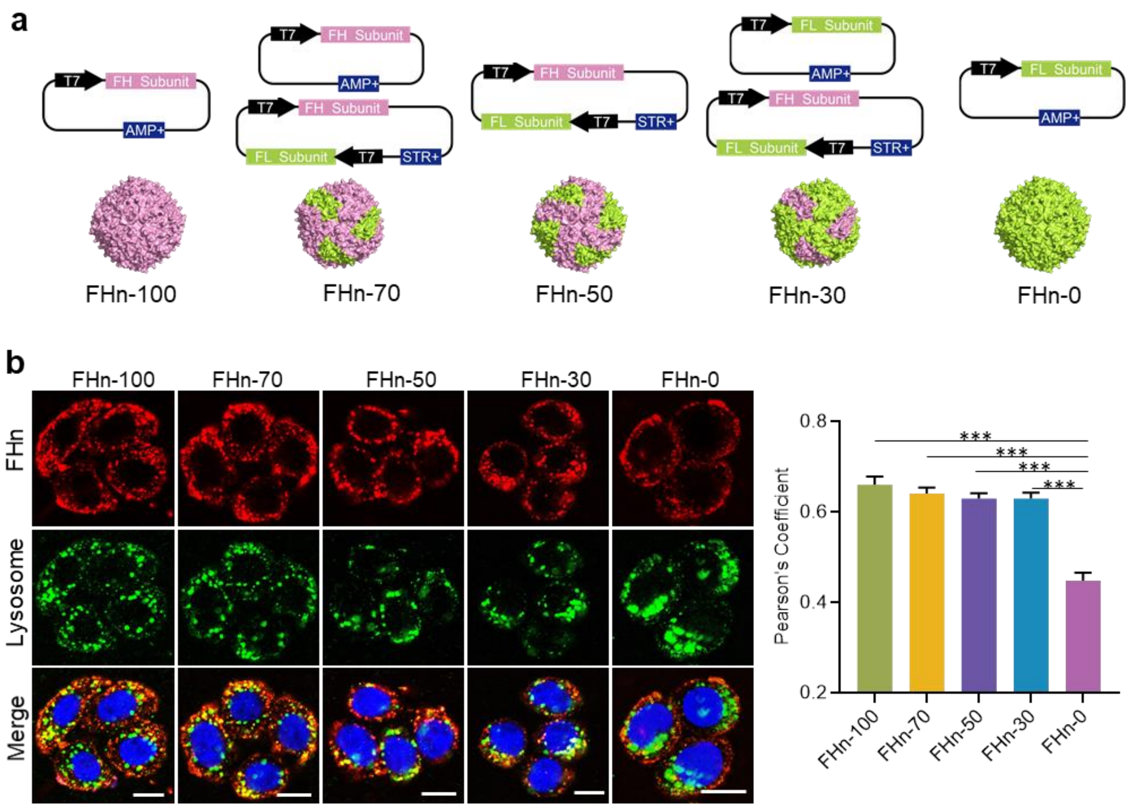

Figure S1. Assembly and intracellular distribution of various FHn. (**a**) Schematic illustration of plasmid compositions of five FHn with different ratios of FH and FL. FHn-100 and FHn-0 were expressed and obtained by transformation of pET 21 expression vector into *E.coli*. The FHn-50 composed of 12 FH and 12 FL needed a bicistronic vector (i.e., pCDFDuet) to co-express the two corresponding genes. The plasmids of FHn-70 and FHn-30 were composed of one pET 21 expression vector and one bicistronic vector, respectively. (**b**) Confocal images and image-based quantification analysis of FHn distribution intracellularly. Blue, nucleus; green, lysosome; red, Cy5-labeled FHn. Scale bar = 10 μm. The co-localization of FHn and lysosomes was determined by Pearson's Coefficient using the Just Another Colocalization Plugin (JACoP) of ImageJ software. ***p<0.001.

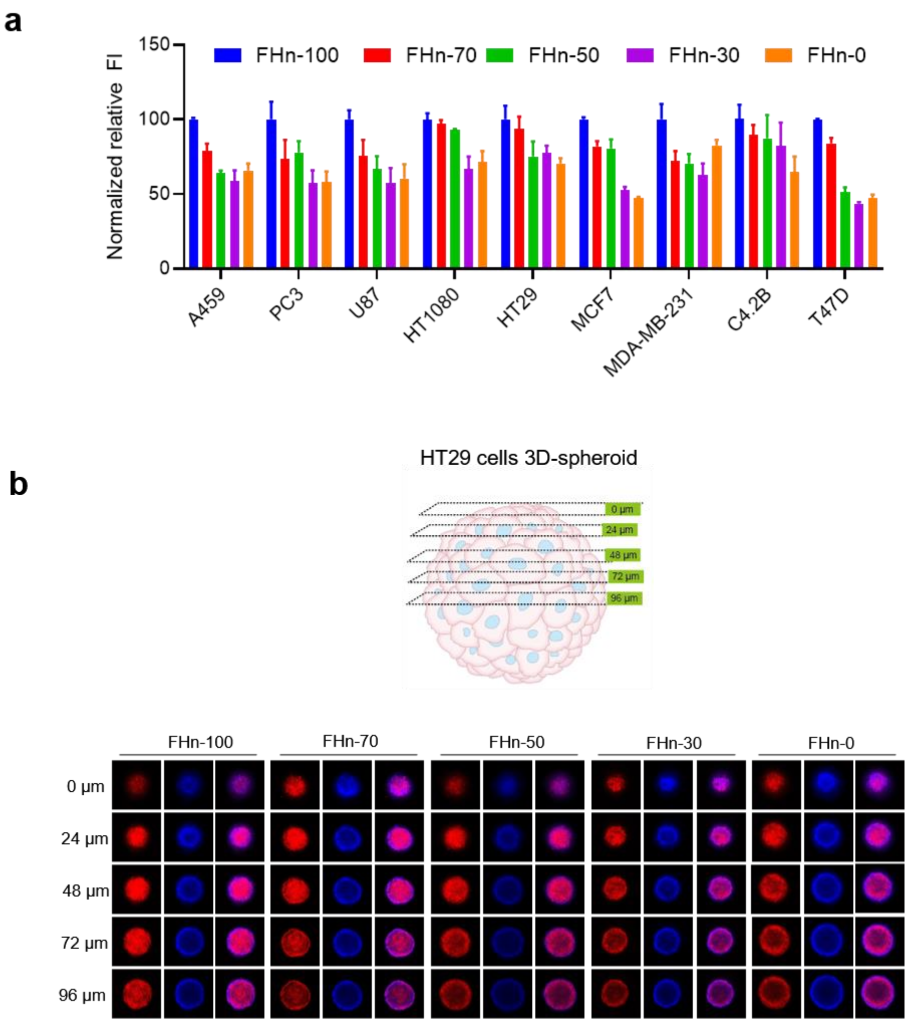

Figure S2. (a) Flow cytometry analysis of cell binding of various FHn in different tumor cells. The uptake of FHn-100 in each tumor cell was normalized to 100. **(b)** Penetration of various FHn in tumor spheroids. Schematic illustration (top) and confocal imaging (bottom) of FHn penetration in 3D tumor spheroids, acquired by a Z-Stack scanning module with 24 μm intervals. Blue, DAPI; red, FHn.

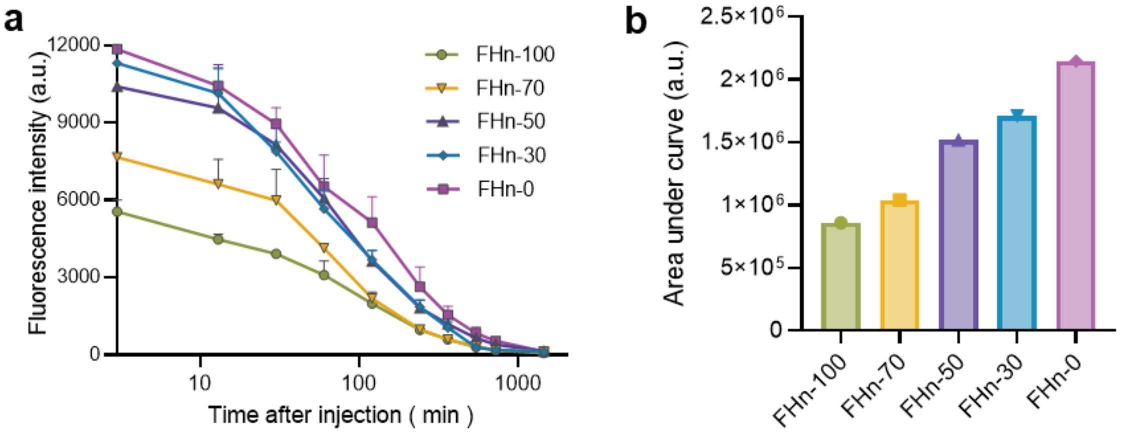

Figure S3. Blood concentration profiles. (**a**) and area under curve (AUC) analysis (**b**) of different FHn over time after intravenous administration (n = 3 per group).

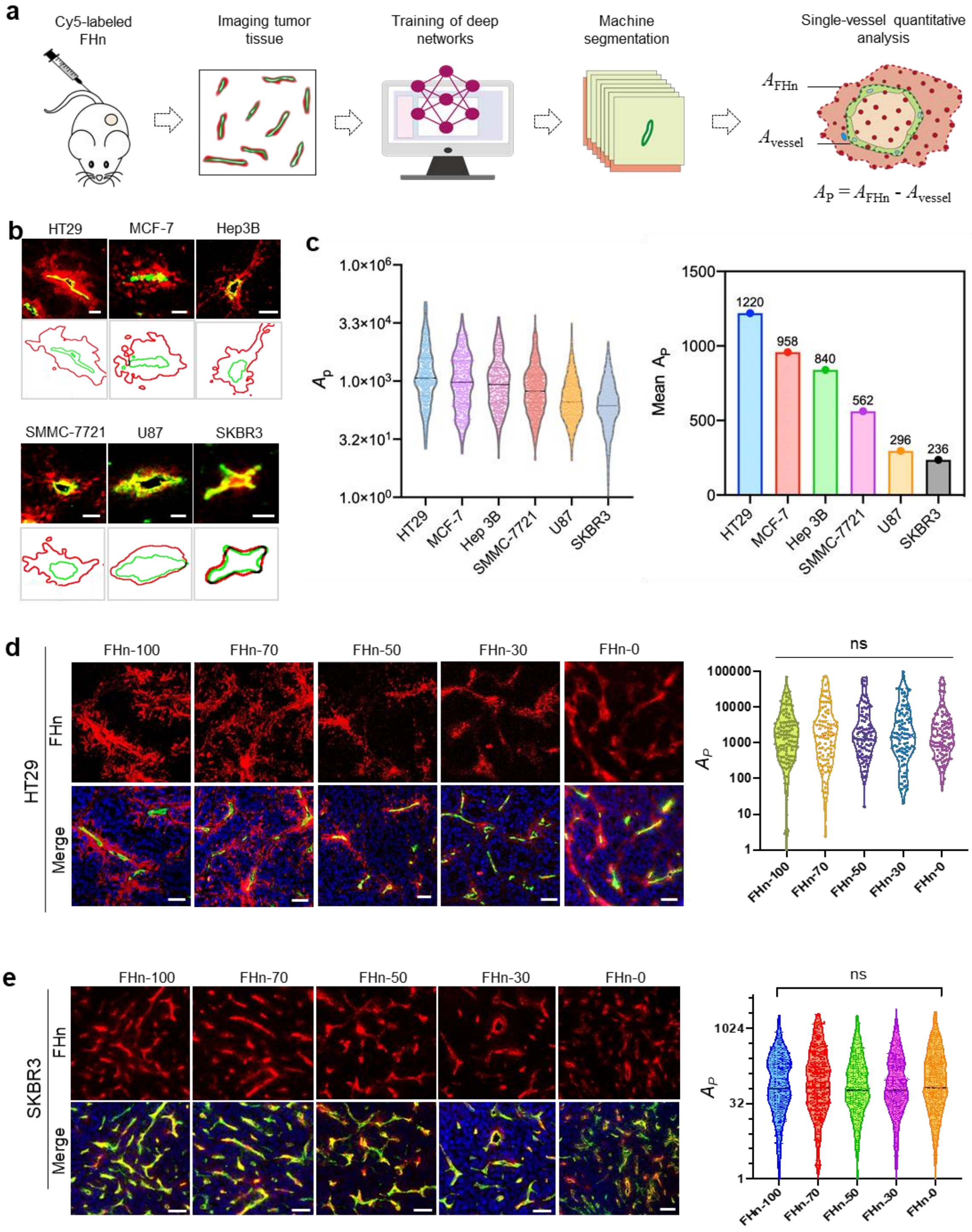

Figure S4. Single-vessel analysis of vascular permeability of various FHn in different tumors. (**a**) Schematic illustration of ML-based single-vessel analysis method. The workflow is composed of multiple steps. The images including tissue distribution of vessel and FHn-based

NPs are firstly acquired following systemic administration of Cy5-labeled NPs. The model for analyzing the images is subsequently trained and established by manually segmentation of the obtained images using a U-Net deep neural network. Finally, the collected images from other samples are automatically segmented using the trained model. According to the automatic segmentation of input images, the vascular permeability of FHn-100 in each vessel was quantitatively assessed by determining the vessel coverage area and FHn-100 penetration area. (**b**) Representative images of vascular penetration of FHn-100 in different tumor models. Top, confocal image for vessel (green) and FHn-100 penetration (red); bottom, the overlay outline of vessel and FHn-100 penetration based on the predicted image from the ML-based model. Scale bar = 50 μm. (**c**) Quantification analysis of vascular permeability using ML-based approach. Left, violin plot analysis of the heterogeneous distribution of A_P for each vessel based on single-vessel analysis method; right, mean A_P of FHn-100 in different tumor types. Each dot represents a single vessel. (**d**) Representative confocal images of vascular penetration of various FHn (left) and violin plot analysis of A_P (right) in HT29 tumors. Scale bar = 50 μm. (**e**) Representative vessels of various FHn away from vessels (left) and violin plot analysis of A_P (right) in SKBR3 tumors. Scale bar = 50 μm.

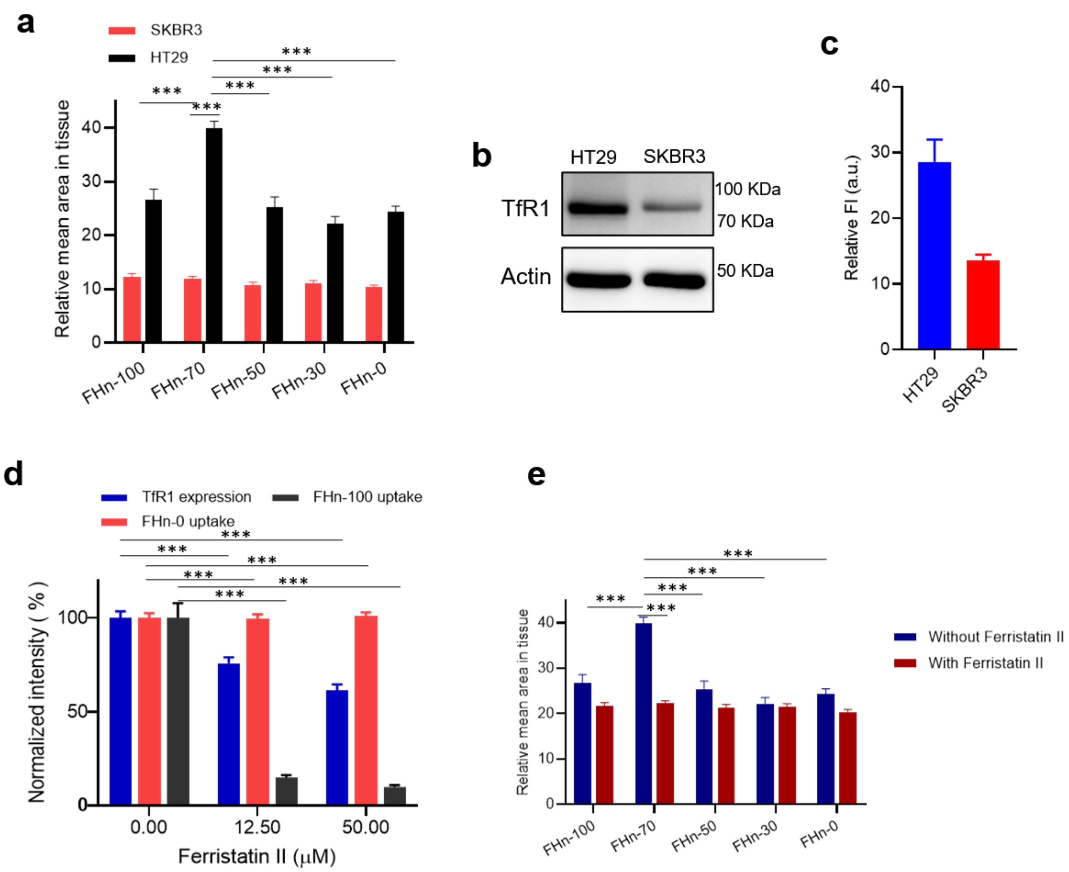

Figure S5. Tumor tissue penetration of various FHn. (**a**) Image-based quantification analysis of mean signal intensity of various Cy5-labeled FHn in tumor tissues. ***p<0.001. (**b**) Western blotting analysis of TfR1 expression in HT29 and SKBR3 cells. (**c**) Flow cytometry analysis of TfR1 expression on the cell membrane surface of HT29 and SKBR3. (**d**)Flow cytometry analysis of TfR1 expression and the uptake of FHn-100 and FHn-0 after pre-treatment with Ferristatin II. ***p<0.001. (**e**) Image-based quantification analysis of tissue penetration of various FHn in HT29 tumors without and with Ferristatin II treatment following intratumor administration. ***p<0.001.

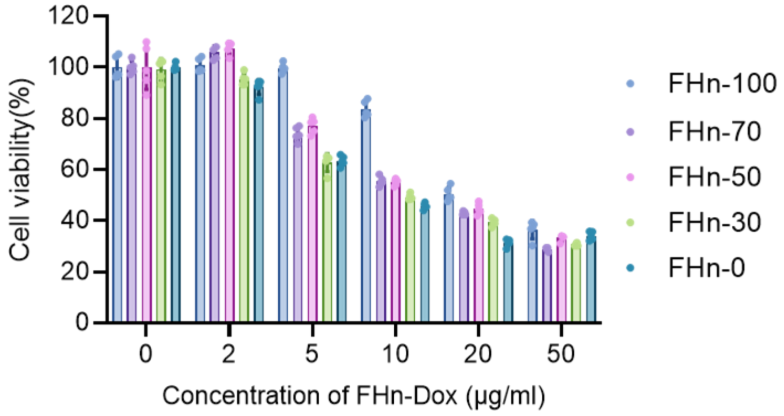

Figure S6. Cytotoxicity assessment of the effect of different FHn-Dox on HT29 cells using CCK8 assay.

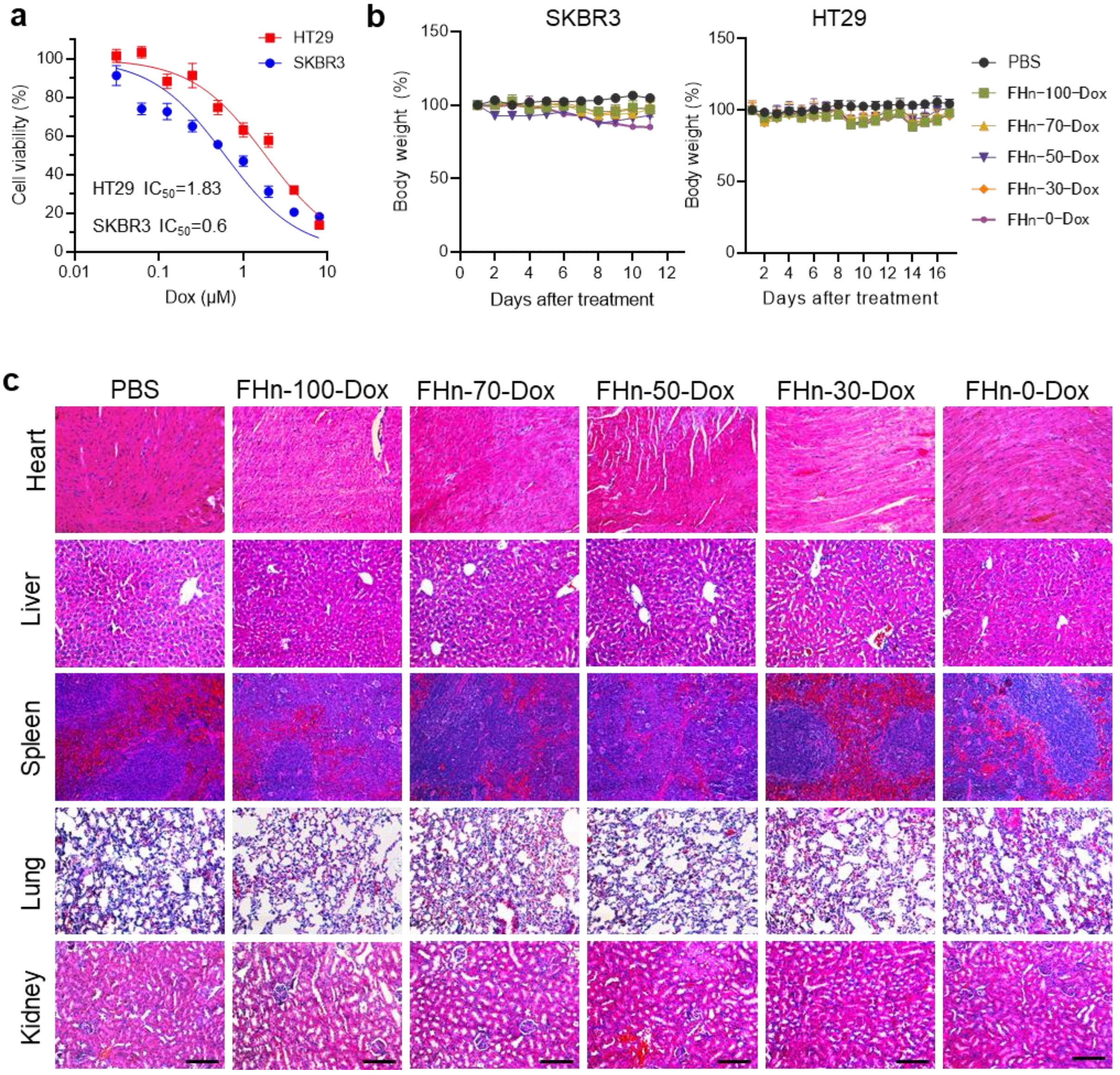

Figure S7. Toxicity assessment of FHn-Dox. (a) The effect of free Dox on cell viability in HT29 and SKBR3 cells. (**b**) The effect of different treatments on body weight of mice. (**c**) H&E staining of the major organs including heart, liver, spleen, lung and kidney collected from different groups. scale bar = 100 μm.

Table S1. Details for different constructs.

Plasmid	ori	Insertion site I		Insertion site II		Constructs
		restriction enzyme	inserted gene	restriction enzyme	inserted gene	
pET21a	ColE1	Nde I, Xho I	FH	-	-	pET21a-FH
pET21a	ColE1	Nde I, Xho I	FL	-	-	pET21a-FL
pET21a	ColE1	Nde I, Xho I	FH-V	-	-	pET21a-FH-V
pCDFDuet	CDF	Nco I, Hind III	FH	Nde I, Xho I	FL	pCDFDuet-FH-FL
pCDFDuet	CDF	Nco I, Hind III	FH	Nde I, Xho I	FH-V	pCDFDuet-FH-(FH-V)
pRSFDuet	RSF	Nco I, Hind III	FH	Nde I, Xho I	FH-V	pRSFDuet-FH-(FH-V)

Table S2. Strategies for different constructs transformation or co-transformation.

Nanocages	Transformed plasmids	Antibiotic resistance
**FHn-100**	pET21a-FH	Ampicillin
**FHn-70**	pET21a-FH and pCDFDuet-FH-FL	Ampicillin and Streptomycin
**FHn-50**	pCDFDuet-FH-FL	Streptomycin
**FHn-30**	pCDFDuet-FH-FL and pET21a-FL	Ampicillin and Streptomycin
**FHn-0**	pET21a-FL	Ampicillin
